# Interactions among Candidate Genes Selected by Meta-Analyses Resulting in Higher Risk of Ischemic Stroke in a Chinese Population

**DOI:** 10.1371/journal.pone.0145399

**Published:** 2015-12-28

**Authors:** Man Luo, Jiaoxing Li, Xunsha Sun, Rong Lai, Yufang Wang, Xiaowei Xu, Wenli Sheng

**Affiliations:** 1 Department of Neurology, First Affiliated Hospital, Sun Yat-Sen University, Guangzhou, Guangdong, China; 2 Department of Neurology, Weifang People’s Hospital, Weifang, Shandong, China; Massachusetts General Hospital, UNITED STATES

## Abstract

Ischemic stroke (IS) is a multifactorial disorder caused by both genetic and environmental factors. The combined effects of multiple susceptibility genes might result in a higher risk for IS than a single gene. Therefore, we investigated whether interactions among multiple susceptibility genes were associated with an increased risk of IS by evaluating gene polymorphisms identified in previous meta-analyses, including methylenetetrahydrofolate reductase (*MTHFR*) C677T, beta fibrinogen (*FGB*, β-FG) A455G and T148C, apolipoprotein E (*APOE*) ε2–4, angiotensin-converting enzyme (*ACE*) insertion/deletion (I/D), and endothelial nitric oxide synthase (eNOS) G894T. In order to examine these interactions, 712 patients with IS and 774 controls in a Chinese Han population were genotyped using the SNaPshot method, and multifactor dimensionality reduction analysis was used to detect potential interactions among the candidate genes. The results of this study found that ACE I/D and β-FG T148C were significant synergistic contributors to IS. In particular, the ACE DD + β-FG 148CC, ACE DD + β-FG 148CT, and ACE ID + β-FG 148CC genotype combinations resulted in higher risk of IS. After adjusting for potential confounding IS risk factors (age, gender, family history of IS, hypertension history and history of diabetes mellitus) using a logistic analysis, a significant correlation between the genotype combinations and IS patients persisted (overall stroke: adjusted odds ratio [OR] = 1.57, 95% confidence interval [CI]: 1.22–2.02, P < 0.001, large artery atherosclerosis subtype: adjusted OR = 1.50, 95% CI: 1.08–2.07, P = 0.016, small-artery occlusion subtype: adjusted OR = 2.04, 95% CI: 1.43–2.91, P < 0.001). The results of this study indicate that the ACE I/D and β-FG T148C combination may result in significantly higher risk of IS in this Chinese population.

## Introduction

Stroke has been recognized as one of the three most common causes of death, and has been established as a major cause of disability in adults[1]. Ischemic stroke (IS), which accounts for 80% of all stroke cases, is multifactorial disorder that may be caused by both genetic and environmental factors as well as their interactions[2]. Increasing evidence from twins, family, and animal model studies has revealed that genetic factors contribute to the pathogenesis of IS[3,4] http://www.sciencedirect.com/science/article/pii/S1474442207700285-bib3#bib3. Additionally, previous studies have identified susceptibility genes that are involved in the development and progression of IS.

Although numerous studies have investigated potential gene polymorphisms in the pathogenesis of IS, and a large number of candidate genes have been proposed, results from these studies have not always been consistent. The lack of consistency in these association and linkage studies may be due to small sample sizes, or a lack of stroke subtype differentiation. Another intriguing explanation is that the loci may contribute to a certain complex disease only by its interaction with other genes, and the main effects of individual loci may be too small to be observed[5]. As IS has been established as a complex multi-gene disease, analyses of the combined effects of multiple genes may be an effective means of providing more detailed information for estimating IS risk.

Meta-analysis has become a powerful tool for candidate gene selection and may be helpful for reconciling the conflicting results seen in loci association studies, and may reduce the uncertainty of disease risk estimates. Previous meta-analyses proposed that the following gene polymorphisms are IS susceptibility gene polymorphisms in the Han Chinese population: methylenetetrahydrofolate reductase (*MTHFR*) C677T, beta fibrinogen (*FGB*, β-FG) A455G and T148C, apolipoprotein E (*APOE*) ε2–4, and angiotensin-converting enzyme (*ACE*) insertion/deletion (I/D)[6]. A more recent meta-analysis also found that the endothelial nitric oxide synthase (eNOS) gene G894T polymorphism was associated with the development of IS in the Chinese population and that the T allele resulted in an increased risk of IS[7]. Therefore, the current meta-analysis–based evidence suggests that the six polymorphisms, namely MTHFR C677T, β-FG A455G, β-FG T148C, ApoE ε2–4, ACE I/D, and eNOS G894T, may be associated with a higher risk of IS in the Han Chinese population.

Multifactor dimensionality reduction (MDR) is a relatively new statistical method and research tool for analyzing gene–gene interactions in complex diseases. We hypothesized that gene–gene interactions among the abovementioned six polymorphisms might result in a higher stroke risk compared to a single susceptibility polymorphism. We tested our hypothesis using MDR in a case–control study in a Han Chinese population. Additionally, as it has been suggested that different IS subtypes may involve different genetic pathophysiological mechanisms[1], we also evaluated genetic marker interactions in the different IS subtypes.

## Materials and Methods

### Study group

This was a hospital-based case-control study. The study design and protocol has been described in detail previously[8,9]. Briefly, 712 IS patients and 774 healthy controls from a Han population in the Guangdong province of China were recruited to this study. A diagnosis of stroke was confirmed after both a careful critical neurological examination and a positive neuroimaging result using either computerized tomography or magnetic resonance imaging according to the International Classification of Diseases, 10th Revision. To determine the stroke subtype, we applied the criteria established by the Trial of Org 10172 in Acute Stroke Treatment (TOAST)[10], and two independent stroke neurologists confirmed our results. The patients were subdivided into the following IS subtypes: 327 with large artery atherosclerosis (LAA), 221 with small-artery occlusion (SAO), 54 with cardioembolism (CE), 52 with other determined etiology (SOE), and 58 with undetermined etiology (SUE). The exclusion criteria for this study were: 1) the presence of other types of cerebrovascular disease (*e*.*g*., intracranial hemorrhage, subarachnoid hemorrhage, transient ischemic attack, cerebral aneurysm, cerebrovascular malformation), and 2) comorbidities with severe systemic diseases such as cancer, severe inflammatory diseases (e.g., rheumatoid arthritis), and serious chronic diseases (e.g., renal failure). Controls that were free from cerebrovascular diseases were selected from local inhabitants, using the same exclusion criteria as for case subjects.

In addition to history of hypertension, diabetes mellitus (DM), dyslipidemia, or ischemic heart disease (IHD), and family history of IS, the following vascular risk factors were also recorded, including age, cigarette smoking habit, body mass index (BMI), systolic blood pressure (BP), diastolic BP, blood glucose, total cholesterol, and triglycerides (TG).

This study was approved by the ethics committee of the First Affiliated Hospital of Sun Yat-sen University (No.[2012]013) and was conducted according to the guidelines of the Declaration of Helsinki. All participants provided informed consent. If participants were unable to communicate, written consent was obtained from their legal guardians. The ethics committee approved this consent procedure.

### Genotyping

Genomic DNA was isolated from a 300-μL blood sample using a TIANamp Blood DNA Kit (TianGen Biotech, Beijing, China) according to the manufacturer’s instructions. The extracted genomic DNA was stored at −80°C until genotyping was performed. We investigated six polymorphisms: five were single-nucleotide polymorphisms and one was an I/D polymorphism (*ACE*). The MTHFR C677T, β-FG A455G, β-FG T148C, ApoE ε2–4, and eNOS G894T polymorphisms were genotyped using the SNaPshot method as described previously[11]. The primers used for the polymerase chain reaction (PCR) are listed in S1 Table. The primers used for the minisequencing extension are listed in S2 Table. Fluorescent-labeled PCR fragments were resolved by capillary electrophoresis on an ABI PRISM 3130xl Genetic Analyzer (Applied Biosystems, Foster City, CA, USA). The data produced were analyzed with GeneMapper 4.0 software (Applied Biosystems). The ACE I/D polymorphism was detected by PCR as described by Rigat and colleagues[12]. The primers used were 5′-CTGGAGACCACTCCCATCCTTTCT-3′ (forward) and 5′-GATGTGGCCATCACATTCGTCAGAT-3′ (reverse). Amplification produced a combination of a 490-bp product and/or a 190-bp product depending on the presence or absence of the ACE insertion allele (I allele), respectively. The amplified products were analyzed by electrophoresis on a 1.5% (w/v) agarose gel (Biowest Agarose, Madrid, Spain) and photographed under UV transillumination.

### Statistical Analysis

Alleles were tested for deviation from Hardy–Weinberg equilibrium (HWE) using a chi-square test. A Student *t*-test was performed to compare age, blood pressure, cholesterol, TG, and glucose levels between groups. A Mann–Whitney U test was used to compare the mean BMI between the case and control groups. The frequency of male gender; cigarette smoking; history of IHD, hypertension, DM, or dyslipidemia; and genotype distributions between cases and controls was compared using a chi-square test. The genetic association was estimated using univariate logistic regression analysis. Statistical analysis of the gene-gene interactions was assessed using MDR. It assigns each cell of a 3×3 interaction table as either high risk or low risk, and then pools these genotype combinations into a two level variable, reducing the dimensionality of the genotypes from N dimensions to one dimension. The one-dimensional genotype variable was evaluated for its ability to classify and predict disease status using a cross-validation (CV) and permutation test[13]. The threshold used to distinguish high-risk and low-risk genotypes was equal to the ratio of cases and controls in the input database. To reduce biased or spurious results due to chance database divisions, we calculated the average CV consistency and prediction error, selecting the model with maximum average CV consistency and minimum average prediction error. Under the null hypothesis of no associations, statistical significance was determined by comparing the average prediction error from the observed data with the distribution of average prediction errors. The null hypothesis was rejected when the P-value derived from the permutation test was ≤0.05. The gene–gene interaction was then determined using logistic regression analysis. Multivariate logistic regression analysis was performed by adjusting for potential confounding factors such as the patient’s age, sex, and their family history of IS, hypertension, and DM. Statistical analysis was performed using SPSS 16.0 statistical software for Windows (SPSS Inc., Chicago, IL, USA) and MDR software (version 2.0 beta 8.4; http://www.epistasis.org/mdr.html). Single-locus analysis was corrected for multiple testing by using Bonferroni-corrected levels of significance (P′ < 0.008). Other tests were two-tailed and a P-value of 0.05 was considered statistically significant.

## Results

### Clinical Characteristics of Participants

The baseline characteristics of the participants are listed in Table 1. The mean age of the controls and cases was 51.5 ± 16.9 years and 65.2 ± 13.9 years, respectively. Men accounted for 54.0% of the controls and 65.3% of the cases of IS. As expected, conventional risk factors for stroke were higher in the IS patients, including advanced age, higher BP, elevated blood glucose, a history of cigarette smoking, and family history of IS, IHD, DM, hypertension, and dyslipidemia.

**Table 1:** Clinical characteristics of IS patients and controls subjects.

	Cases						
	Controls	Total	LAA	SAO	CE	SOE	SUE
	(n = 774)	(n = 712)	(n = 327)	(n = 221)	(n = 54)	(n = 52)	(n = 58)
Age, years	51.5 (16.9)	65.2 (13.9)*	67.4 (12.5)*	65.7 (11.7)*	67.35 (14.02)*	47.4 (15.2)	65.0 (16.2)*
Male, %	54	65.3*	69.1*	61.5	48.1	73.1*	67.2
BMI, kg/m^2^	22.5 (17.7)	23.5 (23.3)	23.9 (18.6)	23.6 (23.3)	22.4 (11.9)	22.2 (12.0)	23.5 (14.3)
SBP, mmHg	125.2 (18.4)	145.4 (22.9)*	148.6 (22.0)*	148.2 (23.6)*	139.24 (20.45)*	127.88 (20.26)	138.42 (19.98)*
DBP, mmHg	77.5 (12.1)	84.3 (14.7)*	85.7 (14.6)*	86.0 (14.6)*	81.57 (17.14)	77.35 (11.76)	78.93 (12.12)
TC, mmol/L	4.83 (1.25)	4.84 (1.30)	4.90 (1.31)	4.85 (1.30)	4.58 (1.24)	4.51 (1.30)	4.96 (1.21)
TG, mmol/L	1.48 (1.0)	1.57 (1.0)	1.56 (0.98)	1.64 (1.03)	1.33 (0.91)	1.49 (0.93)	1.67 (1.36)
Glucose, mmol/L	5.57 (2.03)	5.90 (2.26)*	6.14 (2.56)*	5.61 (1.76)	6.26 (2.38)	5.18 (1.57)	5.91 (2.37)
Cigarette smoker, %		*	*	*		*	
Never	82.4	70.4	62.4	76	85.2	71.2	79.3
Former	1.8	3.7	4.6	4.1	1.9	0	1.7
Current	15.8	26	33	19.9	13	28.8	19
IHD history, %	2.2	7.4*	8.6*	3.2	22.2*	0	10.3*
Hypertension history, %	19	57.3*	66.4*	58.4*	51.9*	15.4	44.8*
DM history, %	7.1	20.2*	21.4*	19.9*	18.5*	5.8	29.3*
Hyperlipidemia history, %	2.6	6.6*	5.2*	8.1*	7.4*	3.8	10.3*
Family history of IS, %	0.6	5.6*	3.4*	8.6*	9.3*	5.8*	3.4*

Ischemic heart disease (IHD), diabetes mellitus (DM), age, systolic blood pressure (SBP), diastolic blood pressure (DBP), and total cholesterol (TC), triglycerides (TG), and glucose values are shown as the mean (standard deviation); body mass index (BMI) is presented as the median (range), and other values as the number of individuals (n) with percentage (n/total N) in parentheses. LAA, large artery atherosclerosis; SAO, small-artery occlusion; CE, cardioembolism; SOE, stroke of other determined etiology; SUE, stroke of undetermined etiology.

*P < 0.05 vs. control.

### Single-locus Analysis

All six polymorphisms followed HWE. Table 2 summarizes the distribution of the different genotypes with regard to overall stroke and the three stroke subtypes. No significant associations were found for all polymorphisms (P′ < 0.008).

**Table 2:** Single-locus genotype distributions of IS and its subtypes.

Genotypes	Stroke				
	Controls	Total	LAA	SAO	CE
	(n = 774)	(n = 712)	(n = 327)	(n = 221)	(n = 54)
**ACE I/D**		0.024*	0.355*	0.024*	0.489*
DD (%)	64 (8.3)	74 (10.4)	32 (9.8)	26 (11.8)	4 (7.4)
ID (%)	351 (45.3)	356 (50.0)	158 (48.3)	114 (51.6)	29 (53.7)
II (%)	359 (46.4)	282 (39.6)	137 (41.9)	81 (36.7)	21 (38.9)
**β-FG T148C**		0.026*	0.069*	0.028*	0.664*
TT (%)	77 (9.9)	29 (8.9)	29 (8.9)	15 (6.8)	4 (7.4)
CT (%)	301 (38.9)	243 (34.1)	106 (32.4)	71 (32.1)	24 (44.4)
CC (%)	396 (51.2)	413 (58.0)	192 (58.7)	135 (61.1)	26 (48.1)
**β-FG A455G**		0.053*	0.084*	0.050*	0.732*
AA (%)	62 (8.0)	46 (6.5)	23 (7.0)	12 (5.4)	4 (7.4)
GA (%)	302 (39.0)	245 (34.4)	107 (32.7)	72 (32.6)	24 (44.4)
GG (%)	410 (53.0)	421 (59.1)	197 (60.2)	137 (62.0)	26 (48.1)
**MTHFR C677T**		0.743*	0.854*	0.866*	0.761*
TT (%)	52 (6.7)	55 (7.7)	25 (7.6)	17 (7.7)	5 (9.3)
CT (%)	299 (38.6)	269 (37.8)	124 (37.9)	86 (38.9)	21 (38.9)
CC (%)	423 (54.7)	388 (54.5)	178 (54.4)	118 (53.4)	28 (51.9)
**ApoE ε2–4**		0.841*	0.396*	0.919*	0.503*
ε2ε2 (%)	3 (0.4)	4 (0.6)	2 (0.6)	1 (0.5)	1 (1.9)
ε2ε3 (%)	107 (13.8)	93 (13.1)	35 (10.7)	36 (16.3)	6 (11.1)
ε2ε4 (%)	8 (1.0)	13 (1.8)	7 (2.1)	2 (0.9)	1 (1.9)
ε3ε3 (%)	535 (69.1)	494 (69.4)	225 (68.8)	152 (68.8)	38 (70.4)
ε3ε4 (%)	113 (14.6)	101 (14.2)	54 (16.5)	28 (12.7)	8 (14.8)
ε4ε4 (%)	8 (1.0)	7 (1.0)	4 (1.2)	2 (0.9)	0 (0.0)
**eNOS G894T**		0.622*	0.577*	0.671*	0.281*
TT (%)	12 (1.6)	14 (2.0)	8 (2.4)	5 (2.3)	0 (0.0)
GT (%)	143 (18.5)	142 (19.9)	62 (19.0)	44 (19.9)	14 (25.9)
GG (%)	619 (80.0)	556 (78.1)	257 (78.6)	172 (77.8)	40 (74.1)

LAA, large artery atherosclerosis; SAO, small-artery occlusion; CE, cardioembolism.

*P-values are based on chi-square test for genotype comparison.

### MDR Analysis

**Fig 1:**
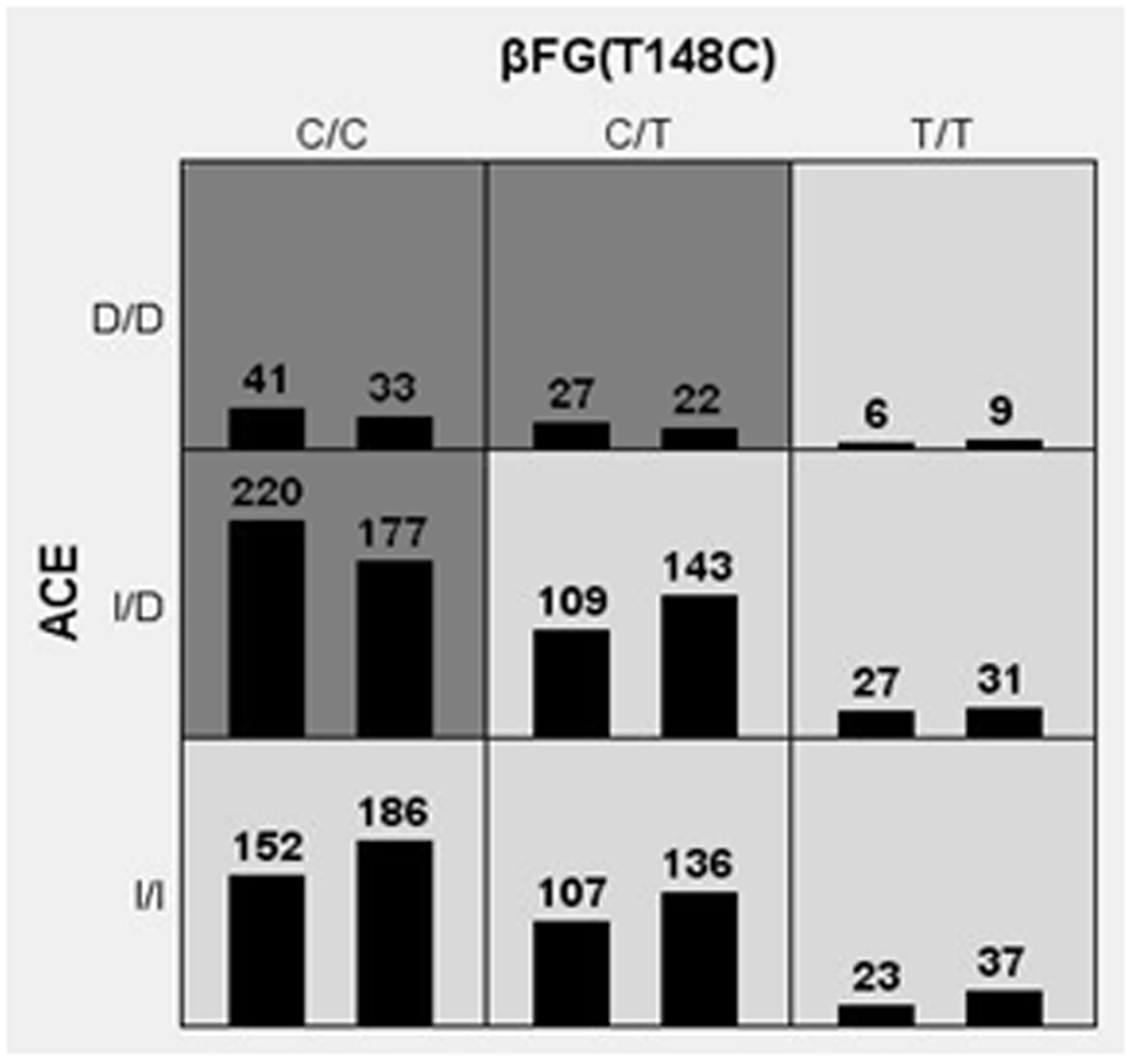
Distribution of ACE I/D and β-FG T148C genotype combinations. ACE I/D and β-FG T148C genotype combinations associated with high risk and with low risk for IS, along with the corresponding distribution of cases (left bars in cells) and of controls (right bar in cells). Dark-shaded cells indicate a high risk of IS (ACE DD + β-FG 148CC, ACE DD + β-FG 148CT, ACE ID + β-FG 148CC combinations); light-shaded cells indicate a low risk of IS (ACE DD + β-FG 148TT, ACE ID + β-FG 148CT, ACE ID + β-FG 148TT, ACE II + β-FG 148CC, ACE II + β-FG 148CT, ACE II + β-FG 148TT combinations).

MDR analysis was used to detect significant interactions between the candidate genes. Table 3 summarizes the CV consistency and testing balance accuracy obtained from MDR analysis in controls and patients with overall IS, for each number of loci evaluated. One 2-order model had a maximum testing balance accuracy of 52.04% (P *<* 0.0001) and the second greatest CV consistency of 9.0 out of 10. This model was composed of the ACE I/D and β-FG T148C polymorphisms, and was selected as the best model. For the LAA and SAO subtypes, the best model was also a 2-order model composed of the ACE I/D and β-FG T148C polymorphisms (LAA: CV consistency = 7/10, testing balance accuracy = 52.66%, P = 0.0008; SAO: CV consistency = 9/10; testing balance accuracy = 56.96%, P *<* 0.0001). MDR analysis was not carried out for the CE subtype because the sample size available for analysis was too small. Fig 1 summarizes the ACE I/D and β-FG T148C genotype combinations associated with high risk and with low risk for IS, along with the corresponding distribution of cases (left bars in cells) and of controls (right bar in cells). Dark-shaded cells indicate a high risk of IS (ACE DD + β-FG 148CC, ACE DD + β-FG 148CT, ACE ID + β-FG 148CC combinations); light-shaded cells indicate a low risk of IS (ACE DD + β-FG 148TT, ACE ID + β-FG 148CT, ACE ID + β-FG 148TT, ACE II + β-FG 148CC, ACE II + β-FG 148CT, ACE II + β-FG 148TT combinations). These data are evidence of gene-gene interaction, as the influence of each specific genotype at a particular locus on IS risk is dependent on the genotype at the other locus.

**Table 3:** Comparison of best models by MDR for overall IS.

No. of loci considered	Best model	Testing balance accuracy	CV Consistency	Sign test (P-value)
1	1^a^	0.5106	6/10	6.941 (0.0084)
2	2, 1	0.5430	9/10	17.889 (<0.0001)
3	6, 2, 1	0.5070	3/10	21.710 (<0.0001)
4	2, 4, 5, 1	0.5204	10/10	42.334 (<0.0001)

^a^ 1–6 denote ACE I/D, β-FG T148C, β-FG A455G, MTHFR C677T, ApoE ε2–4, and eNOS G894T, respectively.

CV, cross-validation.

### Logistic Regression Analysis

According to the results of MDR analysis, we designated the ACE DD + β-FG 148CC, ACE DD + β-FG 148CT, and ACE ID + β-FG 148CC genotype combinations as high risk, and the other genotype combinations as low risk. Logistic regression analysis was used to test the association between the high-risk combinations and risk of stroke. Table 4 depicts the associations between IS and the high-risk combinations as compared with the low-risk combinations. After adjusting for potential confounding IS risk factors (e.g. age, gender, family history of IS, hypertension history and history of diabetes mellitus) using a multivariate logistic analysis, the high-risk combinations had significantly increased risk of IS compared to the low-risk combinations (adjusted odds ratio [OR] = 1.57, 95% confidence interval [CI]: 1.22–2.02, P < 0.001). This interaction appeared more pronounced for the SAO subtype, as the risk of SAO in the high-risk combinations was 2.04 times that of the low-risk combinations (adjusted OR = 2.04, 95% CI: 1.43–2.91, P < 0.001). There was a similar interaction for the LAA subtype in the high-risk combinations as compared to the low-risk combinations (adjusted OR = 1.50, 95% CI: 1.08–2.07, P = 0.016).

**Table 4:** Impact of interaction between ACE I/D and β-FG T148C on IS and its subtypes.

	High-risk combination (%)	Low-risk combination (%)	Adjusted OR* (95% CI)	P-value*
Controls	233 (30.1)	542 (69.9)		
Overall IS	288 (40.4)	424 (59.6)	**1.57(1.22–2.02)**	**<0.0001**
Stroke subtypes				
LAA	129 (39.4)	198 (60.6)	**1.50(1.08–2.07)**	**0.016**
SAO	99 (44.8)	122 (55.2)	**2.04(1.43–2.91)**	**<0.0001**

OR, odds ratio; CI, confidence interval; IS, ischemic stroke; LAA, large artery atherosclerosis; SAO, small-artery occlusion.

*Adjusted for age, sex, family history of IS, hypertension history and history of DM.

## Discussion

Using the MDR method followed by logistic regression analysis, we showed that the ACE I/D and β-FG T148C polymorphisms were significant synergistic contributors to IS and that the ACE DD + β-FG 148CC, ACE DD + β-FG 148CT, and ACE ID + β-FG 148CC genotype combinations resulted in higher risk for both overall stroke as well as the LAA and SAO subtypes.

The human *ACE* gene is located on l7q23 and consists of 26 exons and 25 introns. It has been determined that an I/D polymorphism of the *ACE* gene is related to ACE activity, where the I allele of a 287-bp fragment, and not the deletion allele (D allele), is associated with lower ACE activity[14,15]. As exposure to higher plasma ACE concentrations may result in vascular wall thickness and stiffness, higher plasma ACE concentrations are associated with the risk of cardiovascular and cerebrovascular disease[16]. Generated by ACE, angiotensin II (Ang II) is a powerful vasoconstrictor and a potential proatherosclerotic endogenous compound[17]. Ang II may have proinflammatory effects by stimulating the expression of monocyte chemoattractant protein-1 (MCP-1), which functions by binding to its receptor to induce macrophage accumulation[18]. Thus, ACE can affect BP and accelerate the progression of atherosclerosis.

Fibrinogen (FG) is an important clotting factor and an inflammation marker. FG consists of three pairs of polypeptide chains (Aα/Bβ/Gγ) encoded by the three independent genes for α-FG, β-FG, and γ-FG, respectively. While synthesis of the FG β-chain is the rate-limiting step of FG generation, the *FGB* gene encoding the β-chain is considered the major gene affecting plasma FG levels. A number of previous studies have shown that plasma FG levels may play an important role in the pathogenesis of atherosclerosis[19,20], and certain *FGB* gene polymorphisms are considered a common risk factor for increased plasma FG levels[21,22]. Thus, the β-FG T148C polymorphism may be associated with the pathogenesis of stroke via the regulation of plasma FG levels.

Some studies have shown that interactions among multiple genes may also play a role in the development of IS. A previous study[23] suggested that the β-FG 148CT/TT genotype in combination with the MTHFR 677CT/TT and ACE ID/DD genotypes contributes significantly to susceptibility to IS in the Han Chinese population (OR = 3.907, 95% CI: 1.160–13.162, P = 0.028), but the small sample size comprising 100 patients and 100 controls may have led to unreliable results. Other studies that analyzed different candidate gene profiles concluded that the synergistic effects of multiple genes would result in a higher risk of IS compared to that of single gene polymorphisms[24,25]. However, none of the candidate genes in the above-mentioned studies was selected by meta-analysis, which can avoid randomness of selection.

Many previous studies have examined the association between the ACE I/D or β-FG T148C polymorphism and risk of IS; however, the results were inconsistent. Those genome-wide association studies also did not find an association between the two loci and IS. In our single-locus analysis, no significant associations were found for all polymorphisms, including ACE I/D and β-FG T148C loci. However, our gene-gene interactions analysis found that the ACE I/D and β-FG T148C polymorphisms were significant synergistic contributors to IS. It is possible that the two loci may contribute to IS only by the interaction with each other, and the main effects of individual locus may be too small to be observed.

The precise mechanisms by which the gene–gene interactions between ACE I/D and β-FG T148C affect susceptibility to IS are currently unknown, and additional studies are required to clarify how these two genes may increase IS susceptibility. One possible explanation is that both of these two genes are involved in the pathogenesis and severity of atherosclerosis and inflammation, which are two of the principal pathogenetic factors for IS. Another possible explanation is that gene interactions with marginal effects could lead to increased risk of IS via synergistic effects.

There are some limitations to our study. First, the study population was limited to southern Han Chinese individuals. Thus, our findings require validation in Han Chinese populations from other regions and in other ethnic populations. Second, the population comprised 1486 individuals in total, which is relatively low, however recruiting >1000 individuals from an ethnically homogeneous population is often sufficient for producing reliable data. Third, the study was cross-sectional, and only suggested a simple association. Future studies should investigate the potential causative relations and underlying mechanism.

In conclusion, the present study indicates that the combined ACE I/D and β-FG T148C polymorphisms results in a significantly higher risk of IS, indicating that interactions between these polymorphisms may be synergistically involved in the pathogenesis of IS.

## Supporting Information

S1 Table Primer sequences for PCR. SNP, single-nucleotide polymorphism.
(DOCX)

S2 Table Primer sequences for SNaPshot genotyping. SNP, single-nucleotide polymorphism; SR, SNaPshot reverse; SF, SNaPshot forward.
(DOCX)

S3 Table Relevant data underlying the findings described in manuscript. (XLS)
