# Supplementary material for: Interactions among Candidate Genes Selected by Meta-Analyses Resulting in Higher Risk of Ischemic Stroke in a Chinese Population
Source: PLoS One. 2015 Dec 28;10(12):e0145399. doi: 10.1371/journal.pone.0145399 (PMC4692506; doi:10.1371/journal.pone.0145399)
Supplement: S1 Table — SNP, single-nucleotide polymorphism. (DOCX) [file pone.0145399.s001.docx]

**S1 Table. Primer sequences for PCR**

| Gene | SNP | Primers (forward, reverse; 5′–3′) |
| --- | --- | --- |
| eNOS G894T | rs1799983 | AATGAGGGACCCTGGAGATGAAGTGTTGGGGTGTGGGATCAG |
| β-FG T148C | rs1800787 | AAGGGTCTTTCTGATGTGTATTTTTCATTTTTGGTTCACTTGTTGGCTGA |
| β-FG A455G | rs1800790 | AAGGGTCTTTCTGATGTGTATTTTTCATTTTTGGTTCACTTGTTGGCTGA |
| MTHFR C677T | rs1801133 | TGCAAGTTCTGGACCTGAGAGGAGGCTGACCTGAAGCACTTGAA |
| ApoE ε2–4 | rs429358 | TACAAATCGGAACTGGAGGAACACCCGGCCTGGTACACTGC |
|  | rs7412 | TACAAATCGGAACTGGAGGAACACCCGGCCTGGTACACTGC |

SNP, single-nucleotide polymorphism.
