# Supplementary material for: Interactions among Candidate Genes Selected by Meta-Analyses Resulting in Higher Risk of Ischemic Stroke in a Chinese Population
Source: PLoS One. 2015 Dec 28;10(12):e0145399. doi: 10.1371/journal.pone.0145399 (PMC4692506; doi:10.1371/journal.pone.0145399)
Supplement: S2 Table — SNP, single-nucleotide polymorphism; SR, SNaPshot reverse; SF, SNaPshot forward. (DOCX) [file pone.0145399.s002.docx]

**S2 Table. Primer sequences for SNaPshot genotyping**

| SNP | Sequence |
| --- | --- |
| rs1799983 | SR: TTTTTTTTTTTTTTTTTTTTTTTCAGAAGGAAGAGTTCTGGG |
| rs1800787 | SR: TTTTTTTTTTTTTTAGCAACATCTTCCCAGCAAA |
| rs1800790 | SF: TTTAAACACATTATGATATAACATTACTATTGATTTTAAT |
| rs1801133 | SF: TTTTTTTGCTGCGTGATGATGAAATCG |
| rs429358 | SF: TTTTCGCGGACATGGAGGACGTG |
| rs7412 | SR: GCCTGGTACACTGCCAGGC |

SNP, single-nucleotide polymorphism; SR, SNaPshot reverse; SF, SNaPshot forward.
